# Inflammatory Bowel Disease in Children: Experience and Constraints in a Resource-limited Setting

**DOI:** 10.7759/cureus.7848

**Published:** 2020-04-27

**Authors:** Oluwafunmilayo F Adeniyi, Emuobor Odeghe, Foluke A Olatona, Mary Lawal, Vincent I Onywekwelu, Yeside O Akinbolagbe, Joanah M Ikobah

**Affiliations:** 1 Pediatrics/Gastroenterology, College of Medicine, University of Lagos, Lagos, NGA; 2 Pediatrics/Gastroenterology, Lagos University Teaching Hospital, Lagos, NGA; 3 Gastroenterology, University of Lagos and Lagos University Teaching Hospital, Lagos, NGA; 4 Community Medicine and Primary Care, College of Medicine, University of Lagos, Lagos, NGA; 5 Pediatrics, Lagos University Teaching Hospital, Lagos, NGA; 6 Histopathology, Clina Lancet Laboratories, Lagos, NGA; 7 Pediatrics, College of Medicine, University of Lagos, Lagos, NGA; 8 Pediatrics/Pediatric Gastroenterology, Hepatology and Nutrition Division, University of Calabar/University of Calabar Teaching Hospital, Calabar, NGA

**Keywords:** inflammatory bowel disease, children, resource limited setting, constraints

## Abstract

Introduction: Inflammatory bowel disease (IBD), though well described in the Caucasian population, is rarely encountered in the black African children. The aim of this study was to increase the awareness of this emerging condition in African children and highlight the constraints of management in a resource-limited setting like Nigeria.

Methods: This study included an audit of children with IBD who were seen between January 2015 and February 2020 at the Lagos University Teaching Hospital (LUTH). The clinical records of children aged one to 16 years who presented with recurrent abdominal pain, weight loss, and gastrointestinal (GI) bleeding with clinical suspicion of IBD were reviewed. Clinical features, endoscopic findings, histopathologic findings, and treatment were documented.

Results: Eight children with IBD were seen during the study period. The median age was 12.0 years (range: five to 15 years). The most common reported concerns in the children were chronic abdominal pain [seen in four patients (50%)] and bloody diarrhea [seen in three patients (42.30%)]. Weight loss and arthritis were seen in three (37.5%) and one (12.5%) children, respectively. Endoscopy confirmed two cases of Crohn’s disease (CD), three cases of ulcerative colitis (UC), and three cases of indeterminate colitis (IC). The children with CD were treated with steroids and exclusive enteral nutrition, with one patient receiving methotrexate, while the UC and IC patients received 5-aminosalicylate therapy.

Conclusion: Although IBD is uncommon in Nigeria, a high index of suspicion is vital to enable early diagnosis and appropriate treatment. Management in the African setting is severely constrained by limited access to endoscopy facilities and nonavailability of other effective treatment options such as biologic agents.

## Introduction

Inflammatory bowel disease (IBD) is a disorder of the gastrointestinal (GI) tract characterized by chronic, relapsing, and intermittent inflammation [1], which can affect both adults and children. The spectrum of IBD is composed of Crohn’s disease (CD), ulcerative colitis (UC), and indeterminate colitis (IC) [1]. Diagnosis is made from clinical evaluation, GI endoscopy, and concomitant histopathologic findings [1-2]. Individuals with UC tend to present with recurrent abdominal pain, rectal bleeding, and bloody diarrhea, while patients with CD may present with extraintestinal manifestations such as poor growth, weight loss, musculoskeletal diseases, hepatobiliary diseases, ocular diseases, and renal diseases. Endoscopic findings of UC tend to be more of a uniformly diffuse inflammation of the gut that involves the mucosa and submucosa, while CD typically shows patchy (skip) lesions, transmural gut involvement with or without abscesses, and granulomas [2-3]. The etiology of IBD, though not fully elucidated, is believed to be multifactorial and results from an interplay of genetic predisposition, environmental factors, and immune dysregulation, which ultimately results in chronic inflammation of the gut [2-5].

Pediatric IBD has been well described in the Caucasian and Asian populations [3-7]. Significant variation occurs in the incidence and prevalence of the disease in different countries, while most epidemiological studies report the highest incidence in Europe and North America [8-9]. However, IBD has been sparsely reported in the black population in Sub-Saharan Africa. In Nigeria, Alatise et al. reported 12 cases of IBD in adults from three tertiary facilities in southwest Nigeria in 2012 [10]. In the pediatric age group, there are even fewer reports of IBD in Nigeria. Senbanjo et al. [11] and Ekanem et al. [12] reported five cases of IBD in Nigerian children and UC in a male adolescent, respectively.

Current reports suggest rising global trends in the prevalence of IBD [1, 3, 13-14], and its emergence in developing countries has been attributed to increasing westernization, industrialization, and change in lifestyle and dietary practices. In Nigeria, there are no extensive reviews on IBD in children in terms of clinical presentation, medications, and outcomes. Thus, the aim of this study is to describe the experience from the Lagos University Teaching Hospital (LUTH) in the management of pediatric IBD and highlight the constraints encountered in the management of these patients in a resource-poor country.

## Materials and methods

This study was an audit of cases of IBD seen between January 2016 and February 2020 at the Pediatric Gastroenterology Unit of LUTH following approval from the Health Research and Ethics Review Committee of the hospital and informed consent from parents/patients. LUTH is a 760-bed tertiary facility that receives referrals from within Lagos and its environs.

The diagnosis of IBD was made based on clinical history, physical examination, and endoscopic and histopathologic findings. Extraintestinal clinical features were also documented. Each patient had an upper GI endoscopy and ileocolonoscopy performed by the pediatric gastroenterologist in collaboration with other experienced endoscopists. The Karlz Storz video endoscope (model 13821 PKS/NKS, Germany) was used to perform the endoscopy. Multiple biopsies were obtained during the procedure and sent for histological analysis by experienced pathologists. The European Society of Pediatric Gastroenterology Hepatology and Nutrition [15] criteria for pediatric IBD diagnosis was used to classify study participants. The criteria have sensitivity of 71%-88% and specificity of 77%-89% for CD and sensitivity of 67%-87% and specificity of 89%-97% for UC.

A diagnosis of CD was confirmed by the macroscopic appearance of skip lesions, ulcers, abscesses, and granulomas, and any stricturing or stenosis of the intestine was also noted [15]. No cross-sectional imaging was done for CD. UC, on the other hand, was diagnosed based on macroscopic appearance of diffuse and uniform chronic mucosal inflammation with any appearance of erythema, friability, nodularity and exudates, and exclusive involvement of the colon [15]. IC was reported when the inflammatory changes observed in the colon were not specific or were unclear and did not fit into the typical picture of UC or CD, even after workup was complete.

Baseline investigations, namely full blood count, erythrocyte sedimentation rate (ESR), C-reactive protein, serum proteins, fecal calprotectin, and abdominal scan results were also documented into a standard proforma. The severity of the disease at presentation was also documented with the use of the Pediatric Crohn’s Disease Activity Index (PCDAI) [16] and the Pediatric Ulcerative Colitis Activity Index [17].

Children younger than 18 years were recruited into the study. Results were analyzed using IBM SPSS Statistics for Windows, Version 21.0. (IBM Corp., Armonk, NY). Data were summarized using the median, range, frequencies, and percentages for different variables, including age, sex, clinical features, and laboratory and endoscopic findings.

## Results

Eight patients with IBD were seen during the study period. Six (75.0%) were male patients, and two were female patients (M:F = 3:1) The median age range of the children was 12.0 years (range, five to 15 years). None of the patients had a family history of IBD. Five (62.5%) of the children were from southwestern Nigeria, while the remaining three (37.5%) were from the southern part of the country.

Clinical features

The duration of symptoms before presentation ranged from three months to two years. The main presenting symptoms in all patients were recurrent abdominal pain (75%) and diarrhea with passage of bloody stools (50.0%; Table 1).

**Table 1:** Clinical characteristics, endoscopic and histopathologic findings, and treatment of children with IBD. IBD, inflammatory bowel disease; CD, Crohn’s disease; UC, ulcerative colitis; IC, indeterminate colitis; JIA, juvenile idiopathic arthritis.

Patient	Age (years)	Gender	Clinical presentation	Endoscopic findings	Histologic findings	Treatment	Outcome
1.	5	Male	Chronic abdominal, pain, diarrhea with bloody stools, weight loss, fever	Uniform/continuous hyperemia, ulceration in the rectum, entire colon	UC	Steroids, sulfasalazine	One relapse, doing well
2.	9	Male	Chronic abdominal pain, diarrhea with bloody stools, weight loss, arthritis (JIA), fever	Deep ulcers, cobble stoning, multiple abscesses/purulent exudates and sloughs	CD	Steroids, methotrexate, sulfasalazine	One relapse
3.	14	Male	Recurrent abdominal pain, bloody diarrhea	Uniform/continuous hyperemia, ulceration in the rectum, entire colon	UC	Sulfasalazine	Doing well
4.	9	Male	Recurrent abdominal pain	Multiple aphthous ulcer in the entire colon	CD	Exclusive enteral nutrition	Doing well
5.	15	Male	Recurrent abdominal pain, hematochezia	Hyperemia and loss of vascularity in the descending colon	IC	Sulfasalazine	Doing well
6.	9	Female	Hematochezia	Hyperemia and erosions in the sigmoid and the descending and ascending colon	IC	Commence sulfasalazine	
7.	14	Female	Hematochezia, background nephrotic syndrome		IC	Steroids, commence sulfasalazine	Died
8.	11	Male	Chronic abdominal pain, diarrhea, weight loss, fever	Multiple aphthous ulcers in the descending, transverse colon, areas of hyperemia in same areas, rectal polyp	UC, juvenile polyp	Commence mesalamine	Doing well

Abdominal pain was the most common symptom in the children with CD, while bloody diarrhea was commonly seen in the children with UC and IC, in addition to abdominal pain. The most common extra-GI manifestations seen were weight loss and fever in three (37.5%) of the children, while one (12.5%) had background juvenile idiopathic arthritis (JIA). Another child also had background nephrotic syndrome and had been on steroids; however, she developed lower GI bleeding and subsequently had intestinal perforation.

Laboratory and radiologic findings

One of the children (with background nephrotic syndrome) had severe anemia and had to be transfused. Three of the study participants had evidence of iron deficiency anemia with thrombocytosis, and ESR was remarkably elevated (90 mm/h) in only one of the children who had CD and JIA. The clotting profile was normal in all the children. None of the children had ova or parasites in the stool microscopy and their tuberculosis screenings were negative.

Two of the children had low serum albumin levels, and the serum transaminases were normal in all the children. Fecal calprotectin assay was available in three of the children, and levels obtained were 1,167 (CD), 2,225 (UC) and 2,357 mg/kg (UC), respectively. In the latter child, the assay dropped to 199 mg/kg following one month of treatment.

Only one of the children had a CT scan done, and this revealed thickening of the colon from the cecum to the proximal descending colon.

Colonoscopic and histopathologic findings

**Figure 1:**
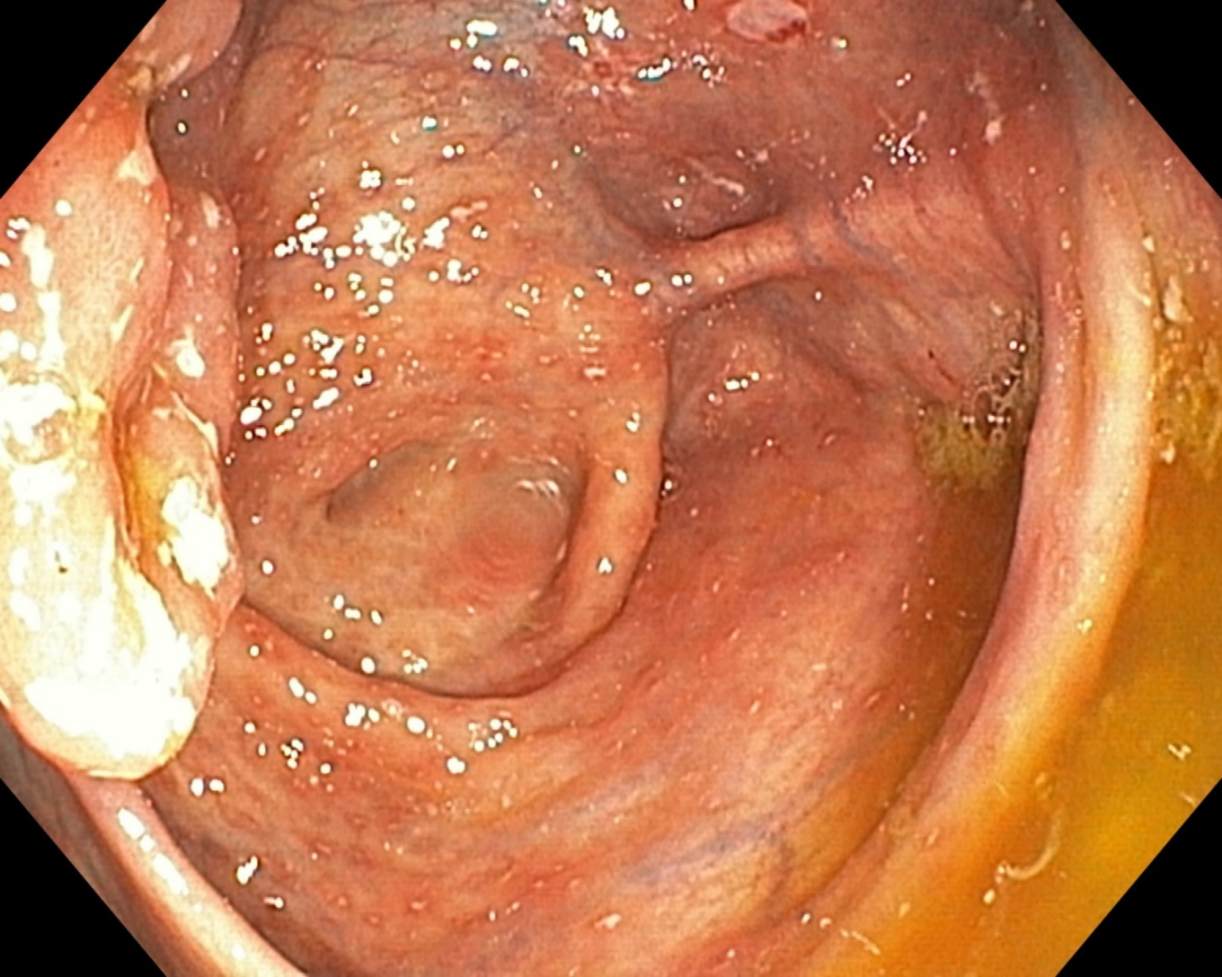
Multiple aphthous ulcers, exudates, and inflamed ileocecal valves in patients with CD. Endoscopic picture CD, Crohn’s disease

**Figure 2:**
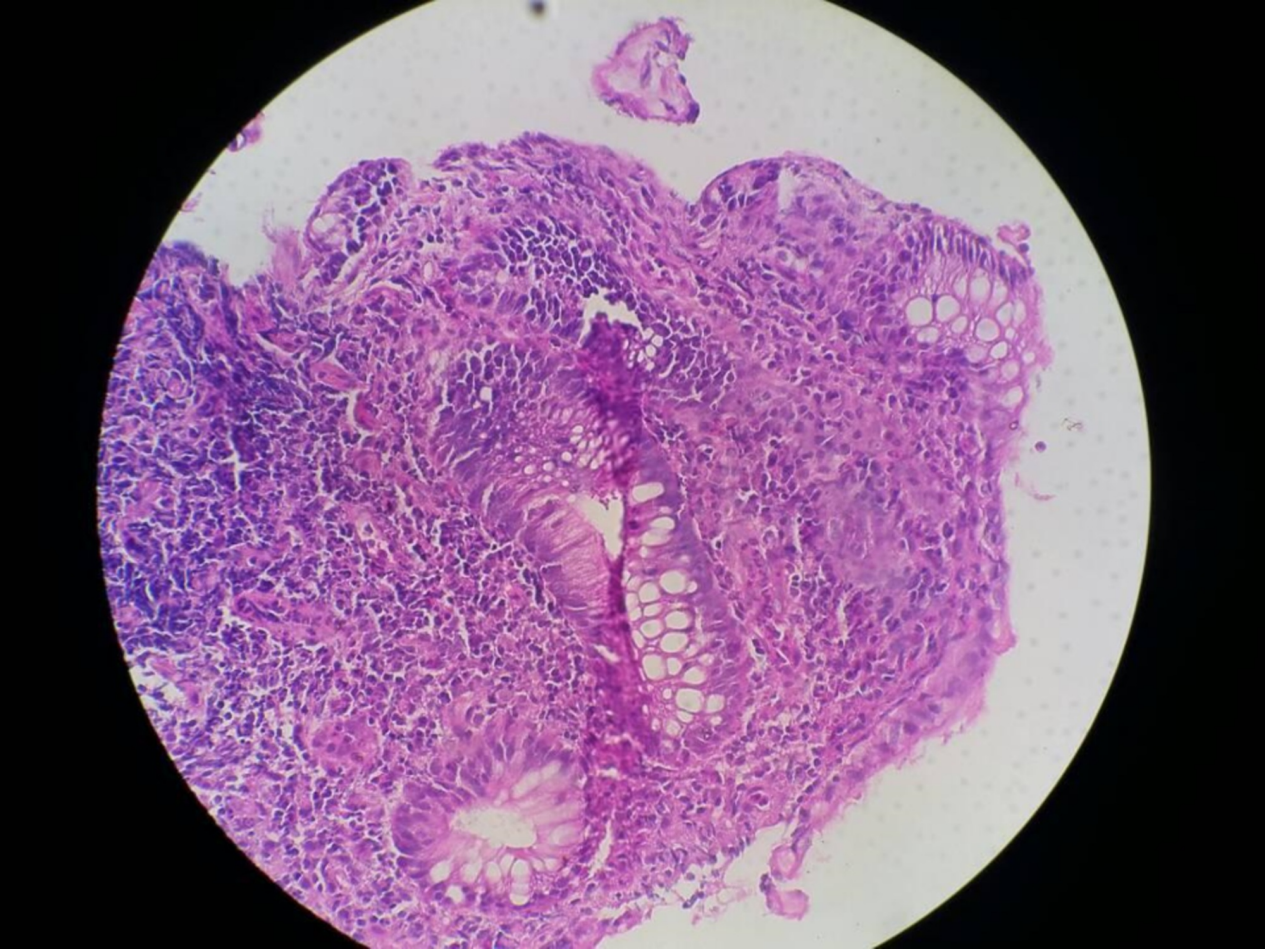
Histology of patients with CD with dense transmural inflammation. CD, Crohn’s disease

**Figure 3:**
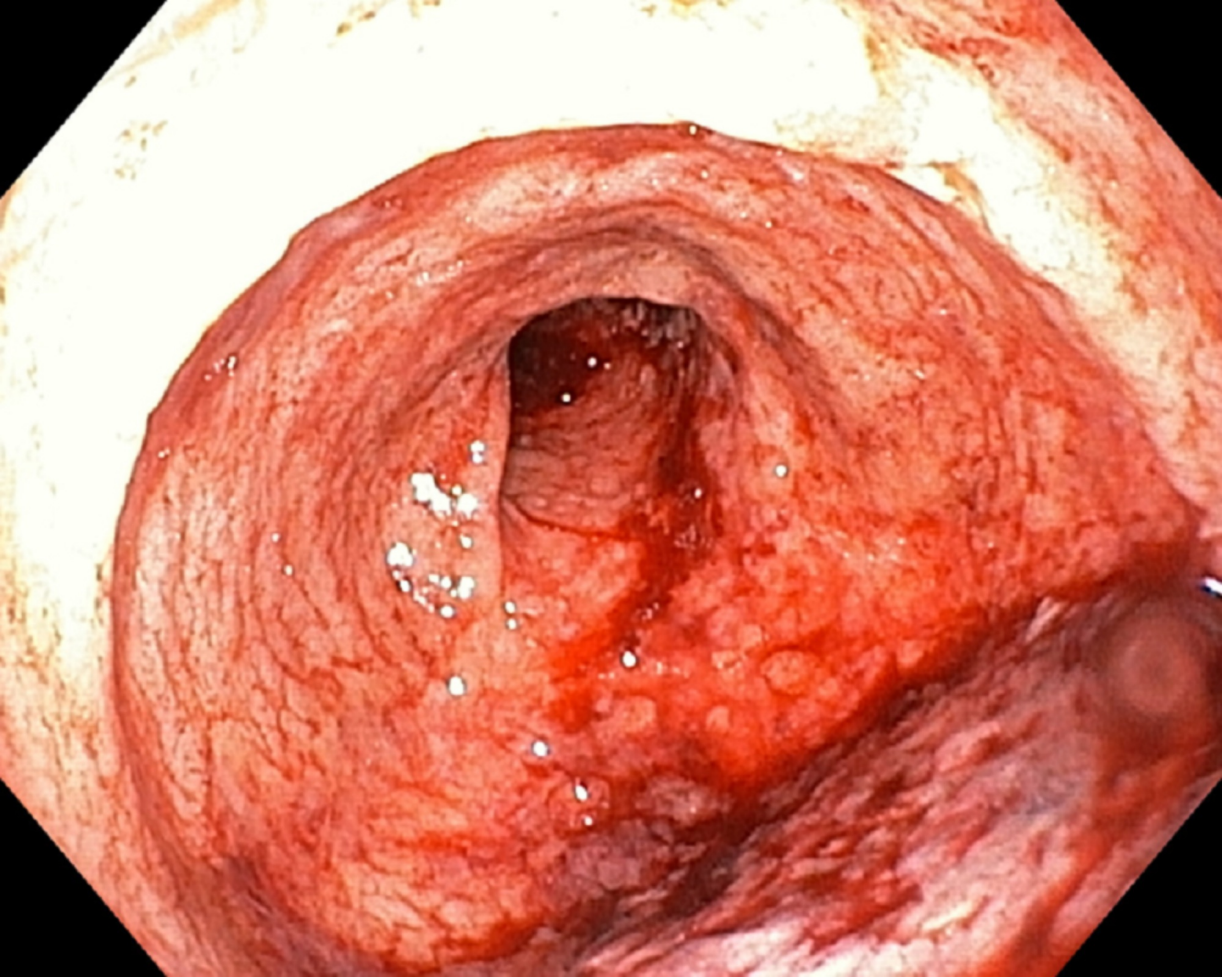
Colonoscopy picture showing uniform continuous inflammation and exudates in patients with ulcerative colitis.

**Figure 4:**
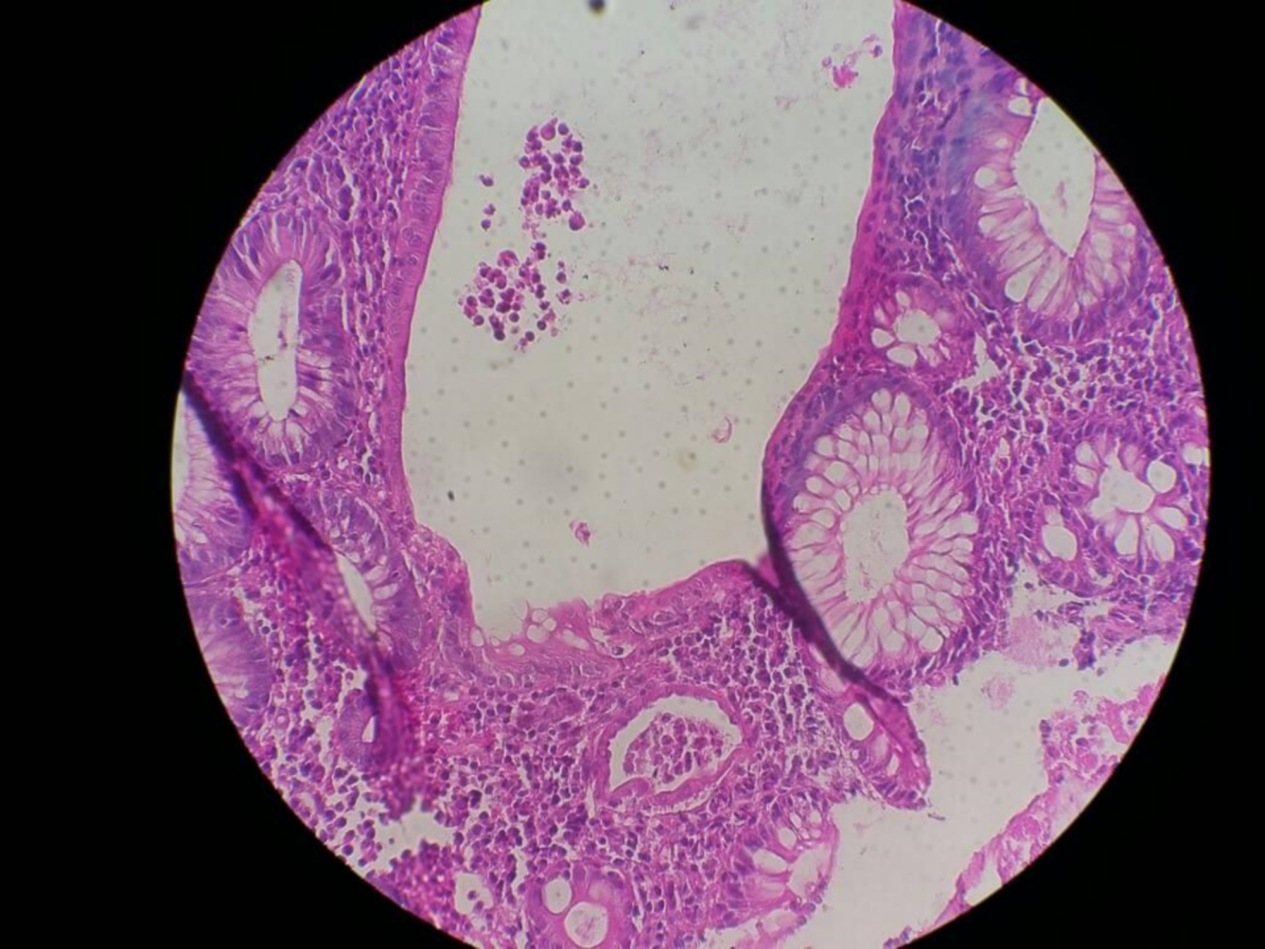
Histology showing crypt abscess in patients with ulcerative colitis.

Two (25.0%) children had colonoscopic and histopathologic features in keeping with CD (Figures 1-2) while three (37.5%) had features in keeping with UC (Figures 3-4; Table 1).

Endoscopic and histopathologic findings in the remaining three (37.5%) children were consistent with IC.

Treatment and outcome

Prior to presentation, the study participants had been treated with various antibiotics and antidiarrheal agents. One of the children with CD had exclusive enteral nutrition for six weeks. The parents were able to procure the formula (modulen® IBD) from outside Nigeria, and subsequently, the child achieved clinical remission. The child with CD and background JIA was on methotrexate before presentation and was to commence a biologic agent (infliximab); however, due to nonavailability of the drug in the country and financial constraints to import the drug, the child was subsequently commenced on steroids and sulfasalazine, and clinical remission was achieved within a month of therapy, but he had a relapse that was subsequently managed with steroids. All the other children with UC and IC received treatment with steroids and 5-aminosalicylates with a good response obtained in most of them, although two children had a relapse from which they recovered following a short course of steroid therapy. The child with background nephrotic syndrome and IC had an intestinal perforation and died a few days postoperatively.

## Discussion

This present study is a comprehensive audit of pediatric IBD in Nigerian children. In this study, eight children were diagnosed with IBD over two years. This is much lower than reports from Europe, North America, and South Africa, and reports on pediatric IBD in West African children are rare, making it challenging to compare pediatric cases across the region [10, 18].

The reasons for this rarity are unclear but were initially attributed to cultural factors. These include infant feeding practices, such as prolonged breastfeeding, reduced rates of antibiotic exposure, overcrowding, sunlight exposure, and exposure to varieties of food that may be contaminated and stimulate gut immunity, thus reducing the risk of IBD [10]. The possibility of under diagnosis due to lack of awareness, lack of appropriate facilities, and trained personnel in many underdeveloped sub-Saharan African countries may also be a possible explanation [10].

In Nigeria, more cases have been reported in the adult population than in the pediatric population. In Zaria (Northern Nigeria), four cases were reported in 2011 by Ukwenya et al. [19], while 12 cases were reported from three tertiary centers in southwestern Nigeria by Alatise et al. [10] in 2012. A 2014 report stated 32 clinico-pathologic cases were reported in the University of Benin Teaching Hospital [20] over four years. Thus, it appears that there is an increase in the number of cases being reported in adults in the country, and this pattern is also being seen in children as well.

The age range of children seen in this present study is from five to 15 years and is similar to reports from Caucasian children [1-2, 8]. It has been reported that CD occurs more commonly in younger children, while UC is reported to be more common during the teenage years and in adults. In our cohort, however, the youngest child had UC. Patients with extreme early onset disease (i.e., onset before the age of six years) have been observed to have more severe disease compared to those with onset after the age of six years [21]. IBD has been rarely described in infants and toddlers; however, a case of UC was recently reported in a 20-month-old child [22]. The clinical presentation of IBD in infants is variable; however, nearly all will have symptoms of chronic diarrhea. Other symptoms commonly reported in infantile IBD include failure to thrive, hematochezia, perianal disease, oral ulcerations, and small bowel obstruction [8]. In contrast to older children and adults, almost all infants with IBD have colonic disease with a higher prevalence of perianal involvement in this age group [8-10].

In terms of gender distribution, there were more male patients in the present study than female patients, and a similar finding was also documented in adults in Benin, Nigeria, by Obaseki and Forae [20]. It is believed that CD is more common in female patients, while an equal sex predilection has been documented in patients with UC. However, reports have been conflicting.

In terms of clinical presentation, two patients in the present study had severe clinical disease (a nine-year-old with CD and a five-year-old with UC). The PCDAI of the patient with CD was up to 50 at presentation. The observation in the UC patient was similar to other reports that document severe disease in young children who present with UC [21-22].

Extraintestinal manifestations of IBD have been described in the literature and include uveitis, scleritis, arthralgia, arthritis, erythema dorsum, pyoderma gangrenosum, and sclerosing cholangitis [1-3, 6]. Extraintestinal manifestations observed in our study included JIA, and a case of IC also had nephrotic syndrome. JIA has been reported in patients with IBD, and in some cases the joint disease may precede the intestinal manifestations, as observed in our study. Renal involvement has also been described in IBD, and these range from glomerulonephritis, nephrolithiasis, tubulointerstitial nephritis, nephrotic syndrome, and amyloidosis. It has been postulated that both conditions may follow a similar pathophysiologic course [23]. There have been a few reports of focal segmental glomerulosclerosis nephrotic syndrome in a patient with UC [24] and CD following mesalamine therapy in patients with nephrotic syndrome [25]. Nevertheless, the causal relationship between the two conditions is still unclear. The presence of these extra-GI manifestations may point to IBD and support its diagnosis.

The diagnosis of IBD in this study was based on clinical features, as well as endoscopic and histopathologic findings. Endoscopic facilities for diagnosis in a country such as Nigeria are still not readily available or affordable, even in the tertiary centers; thus, follow-up colonoscopies to document mucosal healing following therapy remain a challenge. Fecal calprotectin assay (which is a biomarker of gut inflammation and supports the diagnosis of IBD) is largely unavailable and unaffordable in Nigeria, and assays were available in only two of the children with IBD. The assay was remarkably elevated in the five-year-old child with UC (2357 mg/kg) and dropped to 199 mg/kg following one month of treatment. This biomarker measures the excretion of macrophages into the gut, especially when there is chronic inflammation, and the reference levels range from 0 to 50 mg/kg [26-27]. It has high sensitivity and specificity for IBD, is useful for screening and monitoring, and is able to predict IBD relapses [27]. However, the assay is quite expensive in Nigeria, where individuals pay for health services out of pocket; thus, this assay is not affordable, and serial measurements for monitoring purposes are quite difficult. Serological markers have been found to be useful in the diagnosis of IBD and may sometimes help provide clarity in some cases of IC. The perinuclear antineutrophil cytoplasmic antibody is a marker for UC, while anti-Saccharomyces cerevisiae antibodies are commonly positive in cases of CD [28-29].

The children in this present study with UC and IC were treated with sulfasalazine/mesalamine and steroids, which is the usual regimen for these conditions. Other therapeutic options, such as exclusive enteral nutrition, were also employed for those who opted for it and were able to procure it. However, for the case in which a biologic was indicated, the drug was not available and unaffordable for the parents. The child was commenced on sulfasalazine and steroids and went into remission but had a relapse, which was again treated with steroid therapy. Although most of the conventional therapies are useful in the treatment of IBD, the use of biologic agents is more effective, especially for severe disease and perineal disease [2, 4, 18]. However, the use of such agents for the treatment of IBD in a country such as Nigeria, where the payment for medical treatment is still out of pocket, remains out of reach for many families. Thus, there is a need for the government to subsidize therapy for such chronic illnesses.

This study was limited by the fact that it only describes IBD experience in one tertiary center; thus, a multicenter study would enable a more comprehensive description of the condition in the Nigerian environment. A preliminary report (Abstract) of the findings in the study was presented at the 52nd ESPGHAN meeting to create awareness on the occurrence of the disease in black African children [30]. This study illustrates that IBD is increasingly being observed in black African children, which may be a result of improved awareness, as well as increasing availability of diagnostic endoscopic facilities and trained personnel at tertiary centers in developing countries. This finding may also be a reflection of the global increase in the prevalence of IBD, indicating it as an emerging pediatric GI disease in Nigerian children.

## Conclusions

Inflammatory bowel disease is becoming prevalent in black African children, though the diagnosis is being made in the tertiary and specialized centers in developing countries such as Nigeria. Nevertheless, there is a need for physicians to have a high index of suspicion for the condition when children present with chronic abdominal pain and recurrent diarrhea with the passage of bloody stools. Early referral to a gastroenterologist for endoscopic and histopathologic evaluation will enable accurate diagnosis and appropriate intervention.
